# Tuberculosis patients' reasons for, and suggestions to address non-uptake of HIV testing: a cross-sectional study in the Free State Province, South Africa

**DOI:** 10.1186/1472-6963-11-110

**Published:** 2011-05-20

**Authors:** N Gladys Kigozi, J Christo Heunis, Edwin Wouters, Henriëtte S van den Berg

**Affiliations:** 1 Centre for Health Systems Research & Development, University of the Free State, (Nelson Mandela Drive), Bloemfontein, (9300), South Africa; 2 Department of Sociology and Research Centre for Longitudinal and Life Course Studies, Faculty of Political and Social Sciences, University of Antwerp, (Sint-Jacobstraat 2), Antwerp, (2000), Belgium; 3 Department of Psychology, University of the Free State, (Nelson Mandela Drive), Bloemfontein, (9300), South Africa

## Abstract

**Background:**

South Africa endorses the global policy shift from primarily client-initiated voluntary counselling and testing (VCT) to routine/provider-initiated testing and counselling (PITC). The reason for this policy shift has been to facilitate uptake of HIV testing amongst at-risk populations in high-prevalence settings. Despite ostensible implementation of routine/PITC, uptake amongst tuberculosis (TB) patients in this country remains a challenge. This study presents the reasons that non-tested TB patients offered for their refusal of HIV testing and reflects on all TB patients' suggestions as to how this situation may be alleviated.

**Methods:**

In February-March 2008, a cross-sectional survey was conducted amongst 600 TB patients across 61 primary health care facilities in four sub-districts in the Free State. Patient selection was done proportionally to the numbers registered at each facility in 2007. Data were subjected to bivariate tests and content analysis of open-ended questions.

**Results:**

Almost one-third (32.5%) of the respondents reported that they had not undertaken HIV testing, with the most often offered explanation being that they were '*undecided*' (37.0%). Other self-reported reasons for non-uptake included: fear (e.g. of testing HIV-positive, 19.0%); perception of being at low risk of HIV infection (13.4%); desire first to deal with TB 'on its own' (12.5%); and because HIV testing had not been offered to them (12.0%). Many patients expressed the need for support and motivation not only from health care workers (33.3%), but also from their significant others (56.6%). Patients further expressed a need for (increased) dissemination of TB-HIV information by health care workers (46.1%).

**Conclusions:**

Patients did not undergo HIV testing for various patient-/individual-related reasons. Non-uptake of HIV testing was also due to health system limitations such as the non-offer of HIV testing. Other measures may be necessary to supplement routine/provider-initiation of HIV testing. From the TB patient's perspective, there is a need for (improved) dissemination of information on the TB-HIV link. Patients also require (repeated) motivation and support to undergo HIV testing, the onus for which rests not only on the public health authority and health care workers, but also on other people in the patients' social support networks.

## Background

Effective co-management of tuberculosis (TB) and human immunodeficiency virus/acquired immunodeficiency syndrome (HIV/AIDS) necessitates that TB patients undergo HIV testing as early as possible after TB diagnosis [1-3]. In South Africa, provider-initiation of HIV testing for TB patients came into effect in 2007 with the adoption of the provider-initiated testing and counselling (PITC) strategy in the *Tuberculosis Strategic Plan, 2007-2011 *[4]. Findings of a study amongst providers in the Free State Province indicate that almost all nurses (according to their own reports) recommend HIV testing to TB patients, not only at inception of TB treatment (88%), but at every contact (61%) with the patient until acceptance of the test [5]. Despite such commendable efforts, reports indicate that less than half of the registered TB patients accepted HIV testing in 2007 (43.1%) and 2008 (45.9%) [6]. Indeed, at 39%, South Africa achieved a much lower HIV-testing rate amongst TB patients in 2007 than some other sub-Saharan African countries (e.g. Kenya - 79%) also heavily burdened with TB-HIV/AIDS [7].

In April 2010, following limited success of existing HIV-testing policies, the South African government initiated a mass HIV counselling and testing (HCT) campaign aimed at identifying and treating as many eligible persons as possible - TB patients included - before June 2011 [8]. This step confirmed the concerns of government and other interest groups that, despite ostensible implementation of routine/provider-initiated HIV testing, too many TB patients are currently not undergoing HIV testing [1,9]. Research is thus needed to identify obstacles to uptake, and to find ways to improve the rate of HIV testing by TB patients in South Africa. The reported research provides insight from a patient perspective into why routine/provider-initiated HIV testing has been less successful than might have been expected in the case of TB patients. This study follows previous reports on predictors of uptake of HIV testing amongst the same patient population [10,11].

It is important to establish patients' views so as to gain an understanding of non-utilisation of health services [12], which also includes the non-uptake of HIV testing. Evidence from studies conducted among TB populations elsewhere indicates that non-uptake of HIV testing may be attributed to: (1) *patient-/individual-level factors*, including fear of HIV-related stigma, negative experiences during prior HIV counselling, perceived lack of privacy at HIV-testing facilities, and prior HIV testing [13-15]; (2) *relational factors*, such as having to seek sexual partners' consent before undergoing HIV testing [14]; and (3) *health systems factors*, including lack of routine offering of HIV-testing services to TB patients, shortages of HIV testing kits, limited availability of HIV counsellors, poor inter-TB-HIV clinic referral systems, and inadequate counselling skills among providers [16].

However, no research has as yet been conducted to establish patients' reasons for non-uptake of HIV testing in the Free State. Despite a decrease from 32.9% in 2008 to 30.9% in 2009, the province has recorded high rates of HIV prevalence amongst antenatal women since 2007 (range: 30.1% to 32.9%) [17]. Moreover, at 818 cases per 100 000 population in 2007, the province had the fourth highest incidence of TB in the country [18], with six in every ten (60.3%) TB patients testing HIV-positive [6]. Our study sought to identify non-tested TB patients and to explore their reasons for not undergoing HIV testing. We also report both tested and non-tested patients' suggestions regarding what can be done to encourage a greater number of TB patients to undergo HIV testing. The survey formed part of a larger 'fact-finding' research project to inform the development of an intervention to improve uptake of HIV testing by TB patients in the Free State [9].

## Methods

### Setting

Between February and March 2008, we conducted a cross-sectional survey among TB patients in two randomly selected districts, namely Thabo Mofutsanyana and Lejweleputswa. Logistical limitations permitted the purposive selection of only two sub-districts from each district. These were Maluti-a-Phofung and Nketoana from Thabo Mofutsanyana District, and Matjhabeng and Masilonyana from Lejweleputswa District. Primary health care (PHC) facilities in these sub-districts were considered for inclusion in the study if they had provided (1) simultaneous TB and HIV services and (2) TB services to at least ten patients during 2007. Sixty-one out of a total of 97 PHC facilities met these criteria and were thus included in the study.

### Participants

The target population included all registered TB patients aged 18 years or older attending PHC facilities in these specific localities. A sample of 600 patients was considered for the study. The sample size was conservatively estimated from an average of 2 238 patients registered across the 61 PHC facilities during 2007. This was due to a lack of previous studies on uptake of HIV counselling and testing amongst TB patients in the Free State Province to guide statistical sample size calculations. Based on the average number of TB patients registered at each facility during 2007, the proportional-to-size technique was used to determine the number of patients to be recruited at each PHC facility. Patients were conveniently recruited just as they left TB consultation rooms. Although such convenience sampling limits the representativeness of our findings, comparison revealed that the patient sample did not significantly differ from the larger population of TB patients in the four sub-districts under scrutiny in terms of key biographical variables, including sex and age [10].

### Instrument and data collection

A structured interview schedule was developed for data gathering. The schedule, comprising both closed- and open-ended questions, was pre-tested for practicality with ten TB patients at a PHC facility outside the study areas. Information was gathered on *biographical details *(sex, age and education); *clinical aspects *(patient treatment category i.e. whether a patient was undergoing initial [new] or subsequent [re-treatment] TB treatment, treatment duration and HIV testing); *HIV risk-reduction practice *(whether a condom was used during most recent sex); *HIV risk perception *(worry about acquiring HIV, already being infected with HIV or infecting others if already infected with HIV); and *service delivery *(whether the patient received information on the link between TB and HIV at the PHC facility and whether HIV testing was recommended to them).

The open-ended questions assessed: (1) patients' explanations for non-uptake of HIV testing, i.e. '*Please explain why you have never taken a test for HIV*'; and (2) their suggestions as to what could be done to improve their uptake of such, i.e. '*What can be done by health care workers *(*for example doctors, nurses and community health workers*) *in order to encourage more TB patients to test for HIV?*'; and '*What can be done by other people *(*for example family, friends and community*) *in order to encourage more TB patients to test for HIV?*'.

### Data analysis

All closed-ended questions were classified as binary variables: *sex *(male/female); *age *(18-30 years/31 years or older); *patient category *(new/re-treatment); *treatment duration *(60 or less days [intensive treatment phase]/61 or more days [follow-up or continued treatment phase]); *whether a condom was used during most recent sex *(yes/no); *worry about acquiring HIV *(worried/not worried); *worry about already being infected with HIV *(worried/not worried); *worry about infecting others if already infected with HIV *(worried/not worried); *whether patients received information on the link between TB and HIV from the PHC facility *(yes/no); and *whether HIV testing was recommended at the PHC facility *(yes/no). Differences between tested and non-tested patients were examined using chi-square tests.

Content analysis [19,20] was employed in examining responses to the open-ended questions. This approach has been used in research regarding uptake of HIV testing amongst injection drug users [21]. In respect of each open-ended question, four coders used keywords to group similar responses into subcategories for a sample of 25 respondents. After careful consideration and comparison, similar subcategories were combined to form a generic category. The resulting categories were further scrutinised for similarities before compiling a final shortlist of mutually-exclusive categories. One coder then checked all the questionnaires for the frequency of each of the established categories. Categories with very low frequencies were combined to form the category '*other*'. Next, the categories were grouped under the three main themes that emerged from previous studies conducted on uptake of HIV testing amongst TB patients elsewhere [13-15]. These themes related to factors at the: (1) *patient/individual level*, (2) *relational level*, and (3) *health systems/services level*.

### Fieldworker training, ethical clearance and study approval

Ten fieldworkers, four male and six female, were trained in a three-day course entailing lectures on the interview process, i.e. introduction of the interview, mode of asking questions, accurate recording of responses, and the use of probes to seek clarity. To improve consistency in the asking of questions and reporting of answers by all interviewers, the fieldworkers were trained to use the exact wording used in the questionnaire and, in respect of open-ended questions, to record responses verbatim. The fieldworkers further participated in role plays to facilitate their understanding of the questionnaire. Gender issues between interviewers and interviewees were reduced by assigning, where possible, a male and a female interviewer per PHC facility. In most cases, the patients could thus choose whether they wanted to be interviewed by a male or a female interviewer [19].

Patients' participation in the study was voluntary and based on informed consent and guarantee of confidentiality. The fieldworkers explained the study to the patients before requesting their written consent to participate. Patients were assured that their names would only be recorded for purposes of correlating questionnaires with their clinical data and would not appear in any further analysis or reporting of the information.

The Committee for Research Ethics of the Faculty of the Humanities, University of the Free State, approved the study protocol. Permission to conduct the research was granted by the Free State Department of Health at the provincial, district and facility levels.

## Results

### Sample description

All of the 600 patients who were approached agreed to participate in the study. Patients' characteristics stratified by HIV-test uptake are presented in Table 1. Almost one-third (32.5%) of the 600 respondents had not undergone HIV testing. Table 1 shows statistically significant (p < 0.05) differences between HIV-tested and non-tested respondents in respect of sex, patient treatment category, whether a condom was used at most recent sex, whether TB-HIV information was received from PHC facilities, whether the patients were worried about already being infected with HIV, and whether HIV testing had been recommended to them at the PHC facilities.

**Table 1:** Sample description (N = 600)

	Tested for HIV (n = 405)	Not tested for HIV (n = 195)	*P*-value
	
Characteristics	n (%)	n (%)	n (%)
Sex (n = 600)			
Male	175 (43.2)	115 (59.0)	0.00
Female	230 (56.8)	80 (41.0)	
Age in years^‡^(n = 592)			
18-30	218 (54.9)	92 (47.2)	0.07
31 and older	179 (45.1)	103 (52.8)	
Education level (n = 600)			
Primary and lower	145 (35.8)	76 (39.0)	0.45
Secondary and higher	260 (64.2)	119 (61.0)	
Patient treatment category (n = 594)			
New	227 (56.5)	135 (70.3)	0.00
Re-treatment	175 (43.5)	57 (29.7)	
Current TB treatment duration in days (n = 590)			
≤ 60	160 (40.2)	80 (41.7)	0.73
≥ 61	238 (59.8)	112 (58.3)	
Received TB-HIV information from PHC facility (n = 600)			
No	103 (25.4)	111 (56.9)	
Yes	302 (74.6)	84 (43.1)	0.00
Used condom during most recent sex?* (n = 533)			
No	173 (48.5)	104 (59.1)	0.02
Yes	184 (51.5)	72 (40.9)	
Worry will get HIV? (n = 596)			
Not worried at all	185 (45.8)	73 (38.0)	
Worried	219 (54.2)	119 (62.0)	0.07
Worry already have HIV? (n = 596)			
Not worried at all	217 (53.7)	83 (43.2)	0.01
Worried	187 (46.3)	109 (56.8)	
Worry will infect others if already have HIV? (n = 596)			
Not worried at all	189 (46.8)	95 (49.5)	
Worried	215 (53.2)	97 (50.5)	0.54
Recommended for HIV testing? (n = 600)			
No	106 (26.2)	101 (51.8)	0.00
Yes	299 (73.8)	94 (48.2)	

^‡^Mean age = 38.4 years, range = 18-73. *Excludes patients who had never been sexually active or who had not been sexual active in the two months prior to data collection.

Of the patients reporting non-uptake of HIV testing, the majority of respondents were male (59.0%), older than 30 years (52.8%), receiving TB treatment for the first time (70.3%), and had been undergoing treatment for more than two months (58.3%). More than half of non-tested patients had not received information on the link between TB and HIV from their PHC facilities (56.9%), had not used a condom during their most recent sexual activity (59.1%), expressed worry about the possibility that they were already HIV-positive (56.8%), and had not been recommended to undergo HIV testing at the PHC facility (51.8%).

### Non-tested patients' explanations for non-uptake of HIV testing

**Figure 1:**
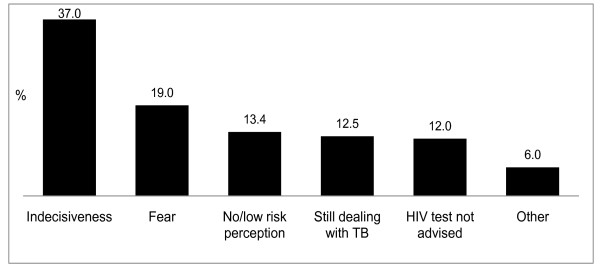
**Patients' reasons for non-uptake of HIV testing (n = 216 citations)**.

Figure 1 reflects non-tested patients' explanations for non-uptake of HIV testing.

### Patient-/individual-level factors

Patient-/individual-level reasons comprised the leading self-reported explanations for patients' non-uptake of HIV testing. Reasons were categorised as '*undecided*' (37.0%) if patients indicated that they were '*still thinking about it*' or '*were not *[yet] *ready*'. All explanations eliciting some form of expressed fear or apprehension were grouped under the category 'fear' (19.0%). These fear-related reasons included fear of HIV testing as such, fear of stigmatisation attached to HIV, and/or fear of the consequences of testing HIV-positive. A further category included patients who reportedly were still grappling with being ill with TB and did not wish to subject themselves to the potential burden of also having to deal with HIV (12.5%), e.g. '*I'm still on TB treatment' *or '*I'm still very sick*'.

### Relational factors

Patients also advanced reasons for non-uptake of HIV testing that reflected the importance of relational factors. Some patients used their sexual partners' HIV-test results as proxy for knowing their own HIV status, e.g. '*I know I am negative because my girlfriend tested and was negative*'. Others stated that they did not need to test because they were cautious or because they trusted their sexual partners, e.g. '*I am always careful*', *'I do not do *[have sex with] *HIV girls*', and *'I trust my *[partner]*'*. Since these patients did not perceive themselves as being at risk regarding HIV infection, their responses were grouped under the category '*no/low risk perception*' (13.4%).

### Health systems-/service-related factors

The most frequent health service-related barrier identified was that some patients had reportedly not (yet) been advised to undergo HIV testing (12.0%), e.g. '*The nurse did not tell me*' and '*No one invited me*'. Others cited service-related explanations (6.0%) like: '*The lay counsellor is always late*', and '*The clinic is always full'*.

### Suggestions towards increasing uptake of HIV counselling and testing by TB patients

Both tested and non-tested patients perceived relational and health systems factors as potential facilitators of HIV-test uptake.

### Relational factors

**Figure 2:**
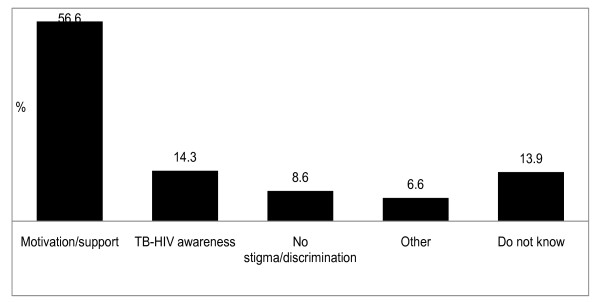
**Patients' suggestions on what other people can do to make HIV testing acceptable to TB patients (n = 698 citations)**.

Figure 2 summarises the TB patients' suggestions as to what other people (e.g. family members, friends and community) can do to make HIV testing more acceptable to them. Most patients' suggestions pointed to a perceived need for motivation or support from others (56.6%), e.g. '*They should stress the importance of HIV testing*', '...*encourage very ill patients to consider HIV testing*', and '*...create support groups*'. The second most frequent category of suggestions implored other people to be more involved in creating awareness around TB and HIV (14.3%), e.g. '*They should read pamphlets to patients*' and '...*involve influential people in TB-HIV education*'. Some suggestions entreated other people to refrain from stigmatising/discriminating against TB patients (8.6%), e.g. '*They should not judge patients*' and '...*should not gossip about patients*'. Suggestions categorised as '*other*' included statements such as '*They should help patients without transport to *[reach] *clinics*' and '...*accompany patients to clinics*'.

### Health systems-/service-related factors

**Figure 3:**
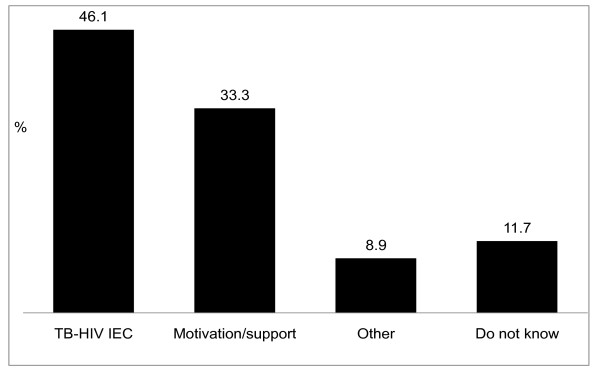
**Patients' suggestions on what health care workers can do to make HIV testing acceptable to TB patients (n = 666 citations)**.

Figure 3 illustrates the TB patients' perceptions of how health care workers (doctors, nurses and community health workers) can facilitate TB patients' uptake of HIV counselling and testing. Overall, the most frequent suggestion was that health care workers should provide patients with (more) information, education and communication (IEC) about (the link between) TB and HIV (46.1%), e.g. '*They should give health talks*', *'*...*inform us about the TB-HIV relationship*' and *'*...*show us pictures of people who are sick *[with AIDS]'.

As was the case with their suggestions regarding the role of family, friends and communities in facilitating uptake of HIV testing, patients also requested service providers to motivate and to be more supportive of TB patients (33.3%), e.g. '*They should emphasise the importance of testing when patients come for treatment*' and '*...form support groups*'. Less frequent propositions (8.9%) included: '*They should test all patients*', '...*should not give *[TB] *treatment without an HIV-test result*', '...*should provide mobile testing*', and '...*provide incentives to test*'.

## Discussion

Almost one-third (32.5%) of TB patients participating in our study had, by their own report, not undergone HIV testing. These patients' reasons for non-uptake of HIV testing mostly amounted to patient-/individual-level factors. It had not been foreseen that many of the untested patients would be '*undecided*' about undergoing HIV testing. However, drawing from previous findings amongst pregnant women in the USA [22] and a quantitative analysis related to the current TB patient population [10] on the significant association between definite HIV-related knowledge and uptake of HIV testing, a possible explanation for the patients' '*indecisiveness*' could be that they were not adequately informed about the TB-HIV link. In line with research in Cambodia [23], most of the total group of tested and non-tested patients in our study suggested that intensifying dissemination of TB-HIV information by health care workers would facilitate their uptake of HIV testing.

Alternatively, given that testing for HIV positions people as being 'at risk' [24], it is possible that patients who were categorised as '*undecided*' actually had no intention of undergoing HIV testing - perhaps for fear of being portrayed as 'promiscuous'. Again, the possibility exists that some of these patients might in fact actually already have undergone HIV testing but did not wish to reveal this, or indeed wished to keep their HIV status secret. Our findings showed a substantial proportion of the patient sample to be worried about either contracting or already having contracted and unknowingly been spreading HIV. This seemed to be a reasonable concern since more than half of all patients by their own report had not used condom protection during their most recent sexual encounter. Hence non-tested patients' supposed '*indecision*' could have been a way to avoid further interrogation from the interviewers.

As with studies amongst TB populations elsewhere [13-15], some patients in our sample had not undergone HIV testing because of fear of HIV testing as such or because of the anticipated consequences of testing HIV-positive, such as being stigmatised. The reality of these patients' fear can be tied to diverse factors, including psychological issues [25], interpersonal relationships [14], and/or socio-structural factors [26]. Following research among TB suspects and patients in Kenya, it was envisaged that the implementation of PITC for TB patients in high HIV-prevalence settings would not only scale up HIV testing, but would also contribute to the mitigation of fears associated with uptake thereof [27]. Yet, as seen in our study, patients' fear of HIV testing persists. By implication, routine/PITC alone may be insufficient towards scaling up HIV testing amongst TB patients. As suggested by patients in our study, there is a need to implement a supplementary broader ethos of motivation and support.

Though research has indicated that TB patients are generally enthusiastic about collaborative TB and HIV activities [28], some patients in our study were nevertheless unwilling to undergo HIV testing, explaining that they still needed to deal with the burden of TB. These patients might have been concerned about the possibility of negative interactions between jointly administered TB and HIV drugs. Indeed, dual administration of TB and HIV treatment may present challenges such as overlapping and additive toxicities [29,30]. However, such potential challenges should be outweighed by evidence demonstrating the feasibility, effectiveness and tolerability of dually-administered TB and HIV therapy [31,32]. There thus seems to be a need to (better) inform patients regarding the advantages of simultaneous treatment for TB and HIV.

Similar to findings of a Cambodian study [33], more than half of non-tested patients in the present study reported that it had not been recommended to them that they undergo HIV testing. The lack of standardisation of clinical records meant that verification of such reports was not possible. However, to the extent that these patient reports are true, such a lack of provider-initiation of HIV testing could have adverse implications for meeting the target of 100% HIV testing by TB patients by 2011 [4]. At the same time, given that it is widely believed and proclaimed that HIV testing should be recommended to all TB patients in high HIV-prevalence settings [2,3,34], the non-practice of provider-initiation of HIV testing raises concerns about health workers' professional ethics and/or training, support and supervision in respect of PITC for TB patients. Our exploratory research suggests that it is imperative for TB control programmes to focus more attention on the implementation of the PITC policy directive.

As far as improving uptake of HIV testing is concerned, most patients asserted that it could be increased by motivation and support of TB patients by both health care workers and significant others in their communities. Also, providing (more) TB-HIV information to patients was perceived to be a salient role for health care providers. Our findings align with evidence from research in Kenya, which established that combined facility and community efforts are feasible and do indeed contribute to the scaling up of HIV testing [27].

Convenience sampling of patients limits the extent to which the findings may be generalised to populations of TB patients beyond the four sub-districts surveyed in the present study. However, as previously mentioned, comparison revealed that the patient sample did not differ significantly from the larger population of TB patients in respect of key biographical variables including sex and age.

Like all research based on self-reports, the current findings are subject to bias. The possibility of interviewer bias such as unintentional errors resulting from interviewers omitting questions or misunderstanding respondents is acknowledged. To reduce such bias we exercised in-field quality control including the immediate editing of questionnaires for completeness and accuracy. Where necessary, patients were traced to provide or clarify missing information. Our study does however offer insight into TB patients' perspectives regarding non-uptake of HIV testing in a specific context.

An important question left unanswered by the current research is that one of the reasons for non-uptake of HIV testing during a current episode of TB might be that patients had been tested for HIV at an earlier stage but due to fear of stigmatisation or other reasons, wished to conceal that and keep their HIV status secret. Our research also did not fully investigate the roles of gender and age. While it was observed that male and older TB patients were less likely to report having been tested for HIV, the reasons for such tendencies and how they can be addressed need to be understood in order to develop appropriate interventions for groups at high risk not to test for HIV.

## Conclusion

Our study found patient-/individual-related factors or personal reasons to figure prominently in TB patients' explanations for non-uptake of HIV testing. This finding contributes to efforts to understand the limited success, to date, of routine/PITC implementation in South Africa. The only health system-related factor that featured - albeit much less prominently - in the patients' accounts of their non-uptake of HIV testing, was the reported non-offer of HIV testing at PHC facilities.

The study findings suggest that from the TB patients' perspective, initiatives to scale up HIV testing by TB patients in the Free State need to consider additional measures to augment routine/provider-initiation of HIV testing. The surveyed patients expressed a need for (more) motivation by and support from both health care workers and significant others, including family, friends and the community. Health care workers also need to (more) consistently and repeatedly inform and educate patients about the link between TB and HIV.

## List of abbreviations used

AIDS: acquired immunodeficiency syndrome; HCT: HIV counselling and testing; PITC: provider-initiated testing and counselling; HIV: human immunodeficiency virus; TB: tuberculosis; VCT: voluntary counselling and testing

## Competing interests

The authors declare that they have no competing interests.

## Authors' contributions

NGK undertook instrument development, fieldwork management, data analysis, initial drafting and revision of the manuscript. JCH was involved in the study design, instrument development, fieldwork management and revision of the manuscript. EW critiqued and contributed inputs towards improvement of the manuscript. HSvdB contributed towards instrument development and commented on the manuscript. All authors read and approved the final manuscript.

## Pre-publication history

The pre-publication history for this paper can be accessed here:

http://www.biomedcentral.com/1472-6963/11/110/prepub
